# Prediction of cancer-associated thrombosis by machine learning: results from the Vienna Cancer and Thrombosis Study

**DOI:** 10.1016/j.esmoop.2026.107764

**Published:** 2026-05-27

**Authors:** T. Hoberstorfer, C.P. Spielvogel, C. Englisch, N. Vladic, S. Nopp, F. Moik, M. Preusser, I. Pabinger, C. Ay

**Affiliations:** 1 Division of Hematology and Hemostaseology, Department of Medicine I, Vienna, Austria; 2 Division of Nuclear Medicine, Department of Biomedical Imaging and Image-Guided Therapy, Medical University of Vienna, Vienna, Austria; 3 Division of Oncology, Department of Internal Medicine, Medical University of Graz, Graz, Austria; 4 Division of Oncology, Department of Medicine I, Medical University of Vienna, Vienna, Austria

**Keywords:** machine learning, neoplasms, pulmonary embolism, venous thrombosis

## Abstract

**Background:**

Improved risk prediction of cancer-associated venous thromboembolism (VTE) remains an unmet clinical need. We aimed to apply machine learning for the prediction of cancer-associated VTE.

**Patients and methods:**

Data from the Vienna Cancer and Thrombosis Study (Vienna-CATS), a prospective cohort study that included patients with cancer from 2003-2019, were used. Patient characteristics and laboratory measurements (including various routine and experimental laboratory assays) were used to train and validate six classification models for VTE prediction. Monte Carlo cross-validation was conducted, with 80% of the samples randomly assigned to the training set and 20% to the test set. Preprocessing algorithms fitted on the training data were applied to the test data to avoid data leakage. Discrimination and input parameter influence were assessed.

**Results:**

In total, 2193 patients (46.6% women) with a median age of 62 (interquartile range 52-68) years were included. The most common tumor types were lung (18.1%), brain (15.3%), and breast cancer (15.1%). Within 6 months and 2 years, 124 (cumulative incidence: 6.4%) and 186 (10.7%) VTE events occurred. The best performing model for VTE prediction was machine learning-enhanced Logistic Regression (LGR) with an area under the curve of 0.66 [95% confidence interval (CI) 0.65-0.67] at 6 months and 0.62 (95% CI 0.61-0.62) at 2 years—comparable to established risk assessment models (RAMs). In LGR, the strongest contributors to VTE risk were homozygous factor V Leiden mutation, rectal, testicular, and pancreatic cancer, newly diagnosed cancer, and history of VTE.

**Conclusion:**

Machine learning models incorporating extensive clinical and biomarker panels did not outperform existing RAMs for cancer-associated VTE.

## Highlights

• Current prediction of cancer-associated VTE is insufficient.
• Machine learning models incorporating extensive clinical and biomarker panels were developed in the Vienna-CATS study.
• Machine learning models did not outperform existing models for cancer-associated VTE.

## Introduction

Venous thromboembolism (VTE) in patients with cancer is a frequent and serious complication that is associated with increased mortality and morbidity.^1^, ^2^, ^3^ Therefore, several risk assessment models (RAMs) have been proposed to predict cancer-associated VTE and to select patients for primary thromboprophylaxis.^4^, ^5^, ^6^ However, these models have demonstrated inconsistent performance in external validation studies, highlighting the need for improved risk prediction of VTE in patients with cancer.^5^

Traditionally, RAMs for cancer-associated VTE have been developed based on statistical methods without the use of machine learning. The most widely recognized model for ambulatory patients with cancer is the Khorana score, which categorizes patients into risk categories based on tumor site and laboratory parameters [hemoglobin, leukocyte count, platelets, and body mass index (BMI)].^7^ Some models developed after the Khorana score have added parameters to improve its performance,^8^, ^9^, ^10^ whereas others have used different sets of input variables to predict VTE.^11^, ^12^, ^13^

Recently, RAMs using machine learning to improve VTE prediction have gained attention. Several models have focused on specific types of cancer and used a small sample size to develop a RAM.^14^ However, the Electronic Health Record Cancer-Associated Thrombosis Risk Assessment Model (EHR-CAT RAM) stands out for being developed using a large database.^15^ Building on the Khorana score, the final model includes six additional variables to enhance VTE prediction. Although it carried out well on electronic health record data during external validation,^16^ its performance was less satisfactory in a prospective cohort study.^17^

Hence, traditional and machine learning RAMs for cancer-associated VTE have been developed, but these models rely on a limited set of readily available clinical variables. In contrast, the predictive value of incorporating experimental parameters remains understudied. The aim of this analysis was to predict VTE in patients with cancer based on a multitude of established and experimental parameters by applying machine learning techniques.

## Patients and methods

### Study design and patient population

We utilized data from the Vienna Cancer and Thrombosis Study (Vienna-CATS), a prospective cohort study including patients with cancer between October 2003 and September 2019, with the main aim of investigating predictive parameters for VTE. The study design and detailed methodology have been reported previously.^18^^,^^19^

Briefly, patients with histologically confirmed newly diagnosed or recurrent cancer were eligible for inclusion. Exclusion criteria included active infection within the preceding 2 weeks, a history of VTE or arterial thrombosis within the past 3 months, and therapeutic dose anticoagulation at the time of enrollment. At study inclusion, medical history and tumor details were collected in a structured interview and by screening of medical records. Results from routine blood draws were recorded, and a blood sample was collected that was used to determine several nonstandard laboratory parameters, that is, biomarker candidates for cancer-associated VTE.^18^^,^^20^, ^21^, ^22^, ^23^, ^24^, ^25^, ^26^ For the current analysis, all patients in the Vienna-CATS study were included.

During the follow-up period, patients were contacted every 3 months by mail or phone to obtain information about the occurrence of VTE. Furthermore, patients were also informed about VTE symptoms at baseline and instructed to report to our hospital at the occurrence of such symptoms. The follow-up period ended after a minimum of 2 years, at the occurrence of VTE or death, loss of follow-up, or withdrawal of consent.

All patients provided written informed consent, and the study was approved by the Ethics Committee of the Medical University of Vienna (EK 126/2003) and conducted according to the principles of the Declaration of Helsinki and its later amendments.

### Outcomes

The primary endpoint of this analysis was VTE, which was defined as incidental, symptomatic or fatal VTE. Any type of VTE was counted as an event, including pulmonary embolism (PE), deep vein thrombosis (DVT) of the legs, catheter-associated VTE, and VTE in unusual locations (i.e. in the cerebral sinus, jugular, upper extremity, caval, portal, or splanchnic veins). Incidentally detected PEs were counted as events if the adjudication committee deemed them to be of clinical significance with a requirement for anticoagulation. Incidental splanchnic vein thrombosis was not considered an event. The diagnosis was objectively confirmed using sonography, venography, computed tomography, or autopsy.

### Statistical analysis

Categorical variables are summarized as absolute frequencies and proportions and continuous variables as median with 25th and 75th percentile [interquartile range (IQR)]. Estimators were obtained with corresponding 95% confidence intervals (CIs).

All baseline variables that were available in >45% of patients were used to predict VTE at 6 months in several machine learning algorithms. Specifically, six classification algorithms were trained and validated for VTE prediction: K-Nearest Neighbor (KNN), Random Forest (RF), Extreme Gradient Boosting (XGB), Explainable Boosting Machine (EBM), Logistic Regression (LGR), and Decision Tree (DT). To ensure accurate performance estimation, 100-fold stratified Monte Carlo cross-validation was conducted, with 80% of the samples randomly assigned to the training set and 20% to the test set. Preprocessing algorithms, including feature selection, hyperparameter tuning, and class balancing [Synthetic Minority Oversampling Techniques (SMOTE)^27^, ^28^, ^29^], were fitted on the training data and then applied to the test data to avoid data leakage. Data leakage occurs when a model has illicit access to information of the test data during the model training process. This can lead to overly optimistic performance estimates and should therefore be prevented.^30^ Missing values were imputed by KNN imputation before feature selection algorithms were applied. Missing values were assumed to be missing at random because many variables in Vienna-CATS were measured within time-restricted subprojects, which resulted in missing data points rather than the variable values themselves. Model performance was assessed by calculating the area under the receiver operating characteristic curve (AUC, C-statistics), accuracy, sensitivity, specificity, positive predictive value (PPV), negative predictive value, and balanced accuracy. The model with the highest AUC was also used in the same manner to predict VTE at 2 years across the entire cohort and to predict VTE in the subgroup of patients with lung cancer.

An analysis was conducted to compare the newly developed machine learning models to established RAMs. Therefore, models for which the required parameters were available in the Vienna-CATS database were selected for performance assessment. The following models met these criteria: the Khorana score,^7^ the Vienna expansion of the Khorana score,^8^ and the Vienna-CATScore.^31^ Based on the available data, use of red blood cell (RBC) growth factors was not included in the calculation of the Khorana score. These models incorporate tumor type to estimate VTE risk; however, risk categories are not defined for all tumor types. Consequently, only patients with tumors for which a risk category was available in the respective model were included in the calculation and a complete case analysis was carried out.

The influence of the input parameters on VTE risk prediction was assessed. For this analysis, the model with the highest AUC (LGR) was evaluated by assessing its coefficients. To relate the importance of the individual features in the machine learning models to their use in classical statistical models, a trait-wide association study (TWAS) was conducted. In this analysis, all parameters were associated with VTE in univariable LGR models with L1 normalization, and coefficients were estimated using maximum likelihood estimation. LGR models were not adjusted for confounders. Parameters were standardized before model fitting. Two-sided *P* values were calculated using the Wald test, and the level of significance was corrected for multiple testing based on the Bonferroni correction, similar to genome-wide association studies.^32^

## Results

### Patient characteristics

In total, 2193 patients in Vienna-CATS were included in the current analysis. The median age was 62 (IQR 52-68) years, the median BMI was 25 (IQR 22-28) kg/m^2^, and 1022 (46.6%) participants were women. The most common tumor types were lung (*n* = 397, 18.1%) and brain (*n* = 336, 15.3%), followed by breast cancer (*n* = 331, 15.1%). Most patients (*n* = 1639, 74.7%) had newly diagnosed cancer, whereas the remaining patients (*n* = 554, 25.3%) had recurrent disease. A history of VTE was recorded in 103 (4.7%) patients and 181 (8.3%) had a positive family history for VTE. Detailed patient characteristics are displayed in Table 1.

**Table 1:** Patient characteristics

Characteristic	Missing	Value
Age (years)	0 (0%)	62 (52-68)
Sex	0 (0%)	
Male		1171 (53.4%)
Female		1022 (46.6%)
BMI (kg/m^2^)	9 (0.4%)	25.0 (22.0-28.0)
Smoking	356 (16%)	
Never		915 (49.8%)
Current		583 (31.7%)
Previous		339 (18.5%)
History of VTE	0 (0%)	103 (4.7%)
Family history of VTE	0 (0%)	181 (8.3%)
Arterial hypertension	0 (0%)	740 (33.7%)
Diabetes	0 (0%)	198 (9.0%)
CVD	0 (0%)	222 (10.1%)
Tumor type	0 (0%)	
Lung		397 (18.1%)
Brain		336 (15.3%)
Breast		331 (15.1%)
Lymphoma		271 (12.4%)
Colon		199 (9.1%)
Prostate		161 (7.3%)
Pancreas		156 (7.1%)
Other		342 (15.6%)
Diagnosis status	0 (0%)	
First diagnosis		1,639 (74.7%)
Recurrence		554 (25.3%)
TNM staging	1012 (46%)	
IV		626 (53.0%)
II		214 (18.1%)
III		185 (15.7%)
I		151 (12.8%)
0		5 (0.4%)
Leukocyte count (G/l)	9 (0.4%)	7.2 (5.7-9.6)
Platelet count (G/l)	9 (0.4%)	250 (199-310)
Antithrombin (%)	55 (2.5%)	105 (94-115)
Prothrombin fragment 1 + 2 (pmol/l)	58 (2.6%)	226 (165-320)
Soluble P-selectin (ng/ml)	36 (1.6%)	38 (29-49)
D-dimer (mg/l)	92 (4.2%)	0.71 (0.37-1.44)
Growth differentiation factor 15 (pg/ml)	662 (30%)	1,004 (654-1,752)
		*N* = 2193

TNM staging is only applicable to solid non-brain cancers.

BMI, body mass index; CVD, cardiovascular disease; VTE, venous thromboembolisml; TNM, tumor–node–metastasis.

### Thromboembolic events

Within 6 months of follow-up, 124 (cumulative incidence 6.4%, 95% CI 5.3% to 7.4%) VTE events were detected. Of these, 63 (50.8%) were PEs, of which 7 (5.6%) were fatal. Regarding DVTs, 43 (34.7%) occurred in the legs, 8 (6.5%) in the upper extremity, 4 (3.2%) in the splanchnic veins, and 3 (2.4%) in the jugular veins. One thromboembolic event each was a catheter-associated VTE, a cerebral sinus vein thrombosis, and a thrombosis in the superior vena cava. During the entire observation period of 2 years, 186 VTE events occurred (cumulative incidence 10.7%, 95% CI 9.2% to 12.2%).

### Model performances

Of 92 total available variables in Vienna-CATS, 18 were removed because they had >55% missing values. The remaining 74 variables (35 clinical and 39 biomarkers) were used as input for the machine learning models before preprocessing (Supplementary Table S1, available at https://doi.org/10.1016/j.esmoop.2026.107764). The performance of the newly developed machine learning models for VTE prediction at 6 months is displayed in Table 2 and Supplementary Figure S1, available at https://doi.org/10.1016/j.esmoop.2026.107764. LGR yielded the highest AUC at 0.66 (95% CI 0.65-0.67), followed by RF, EBM, and XGB with AUCs >0.6, indicating moderate performance, whereas KNN and DT showed poor AUCs <0.6. All models demonstrated low sensitivity (up to 52% in LGR), whereas specificity was moderate to very high (between 69% in LGR and up to 98% in XGB). PPVs were generally very low, but negative predictive values remained high in all models (>94%). The accuracy of the models was mostly high (between 68% for LGR and 93% for XGB) but decreased substantially when the balanced accuracy was calculated instead (highest in LGR at 60%). The performance analysis was then repeated with the model with the highest AUC (LGR) to predict VTE at 2 years (Table 2 and Supplementary Figure S2, available at https://doi.org/10.1016/j.esmoop.2026.107764). Herein, a similar pattern of performance metrics was observed. In the subgroup of patients with lung cancer, LGR showed performance metrics comparable to the results of the entire cohort (Supplementary Table S2, available at https://doi.org/10.1016/j.esmoop.2026.107764).

**Table 2:** Model performances for venous thromboembolism prediction

Model	Accuracy	Sensitivity	Specificity	PPV	NPV	BAcc	AUC
**180 days**							
KNN	0.79 (0.79-0.79)	0.24 (0.23-0.25)	0.82 (0.82-0.83)	0.08 (0.07-0.08)	0.95 (0.95-0.95)	0.53 (0.53-0.54)	0.55 (0.54-0.56)
RF	0.90 (0.90-0.90)	0.09 (0.08-0.10)	0.95 (0.95-0.95)	0.10 (0.09-0.11)	0.95 (0.94-0.95)	0.52 (0.52-0.52)	0.63 (0.62-0.64)
XGB	0.93 (0.93-0.93)	0.04 (0.03-0.05)	0.98 (0.98-0.98)	0.11 (0.10-0.13)	0.94 (0.94-0.94)	0.51 (0.51-0.51)	0.61 (0.61-0.62)
EBM	0.82 (0.81-0.82)	0.27 (0.26-0.29)	0.85 (0.85-0.86)	0.10 (0.10-0.10)	0.95 (0.95-0.95)	0.56 (0.56-0.57)	0.63 (0.62-0.63)
LGR	0.68 (0.68-0.68)	0.52 (0.51-0.54)	0.69 (0.69-0.69)	0.09 (0.09-0.09)	0.96 (0.96-0.96)	0.60 (0.60-0.61)	0.66 (0.65-0.67)
DT	0.75 (0.74-0.76)	0.28 (0.26-0.30)	0.78 (0.77-0.79)	0.07 (0.07-0.08)	0.95 (0.95-0.95)	0.53 (0.52-0.54)	0.54 (0.53-0.55)
**730 days**							
LGR	0.65 (0.65-0.65)	0.50 (0.49-0.51)	0.66 (0.66-0.67)	0.12 (0.12-0.12)	0.94 (0.93-0.94)	0.58 (0.58-0.59)	0.62 (0.61-0.62)

Numbers in parentheses represent the 95% CI.

AUC, area under the curve (C-statistics); BAcc, balanced accuracy; CI, confidence interval; DT, Decision Tree; EBM, Explainable Boosting Machine; KNN, K-Nearest Neighbor; LGR, Logistic Regression; NPV, negative predictive value; PPV, positive predictive value; RF, Random Forest; XGB, Extreme Gradient Boosting.

### Comparison with established models

Table 3 shows a comparison between the best performing newly developed machine learning model (LGR) and established RAMs. The AUC of all models (except the Khorana score) was in the moderate range, demonstrating comparable discrimination of machine learning and established RAMs.

**Table 3:** Comparison of discrimination to established RAM

Model	AUC	*N*
LGR	0.66 (0.65-0.67)	2193
Khorana score	0.58 (0.52-0.64)	1776
Khorana score—Vienna expansion	0.65 (0.60-0.71)	1989
Vienna-CATScore	0.67 (0.61-0.73)	1645

Numbers in parentheses represent the 95% CI.

Comparison of the best performing machine learning model (LGR) to established RAMs. LGR model was applied to the entire cohort, whereas each RAM was evaluated in the subset of patients to which it is applicable.

AUC, area under the curve; CI, confidence interval; LGR, Logistic Regression; RAM, risk assessment model.

### Influence of input parameters

**Figure 1:**
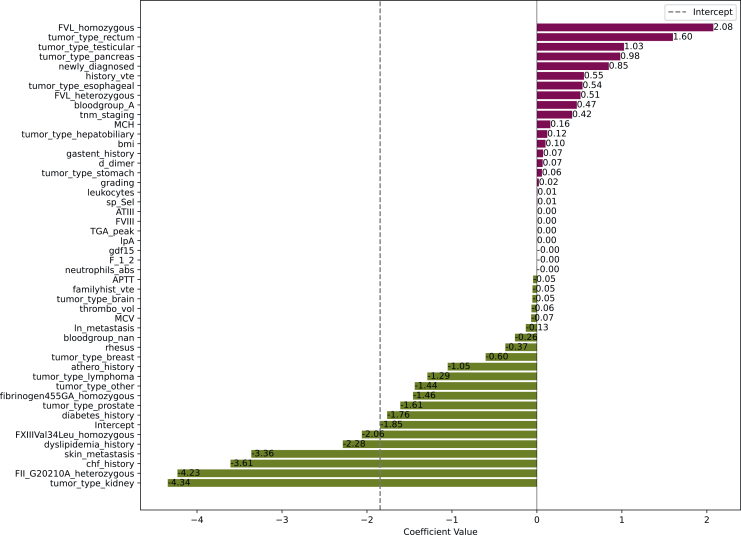
**Coefficients of prediction variables in LGR for the prediction of cancer-associated thrombosis at 6 months.** Burgundy bars represent positive associations, and green bars represent negative associations. LGR, Logistic Regression.

In the LGR model, 47 variables were included to predict VTE after preprocessing. The strongest positive association of input parameters in LGR was demonstrated by homozygous factor V Leiden mutations and the tumor types rectal, testicular, and pancreatic, whereas the tumor type kidney, prothrombin mutation, and chronic heart failure showed the strongest negative association (detailed in Figure 1). This pattern was similar in the VTE prediction at 2 years (Supplementary Figure S3, available at https://doi.org/10.1016/j.esmoop.2026.107764).

**Figure 2:**
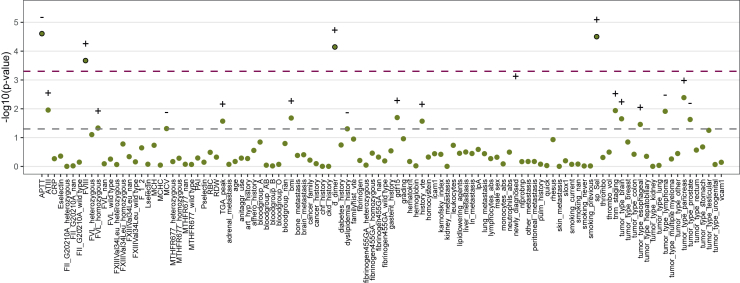
**TWAS of all input parameters with venous thromboembolism at 6 months.** Green dots represent the *P* value, +/− represent positive/negative associations, and the dashed lines represent the uncorrected (gray) and corrected (burgundy, Bonferroni) significance thresholds. APTT, FVIII, D-dimer and sP-selectin showed the strongest positive/negative association with VTE. APTT, partial thromboplastin time; FVIII, factor VIII; sP-selectin, soluble P-selectin; TWAS, trait-wide association study.

In a comparative TWAS analysis using classical statistical methods, a significant positive association with VTE at 6 months was found for factor VIII activity, D-dimer, and soluble P-selectin (sP-selectin), whereas activated partial thromboplastin time was negatively associated (Figure 2).

## Discussion

This study investigated the prediction of cancer-associated VTE in a cohort including various established and experimental parameters. In total, 74 (35 clinical, 39 potential biomarkers) parameters were used as input to train and validate machine learning models. Model performance varied between poor and moderate, with the best performing model being LGR, with an AUC of 0.66 (95% CI 0.65-0.67). Specificity and negative predictive values remained high for the models, whereas sensitivity and PPVs were low. The discriminatory performance of machine learning prediction models was similar to that of established RAMs. Furthermore, secondary analyses were conducted with the LGR model, confirming its performance in a 2-year analysis and in the subgroup of patients with lung cancer. A TWAS showed the differences in input parameter relevance between traditional statistical and machine learning approaches.

Several machine learning models for the prediction of cancer-associated VTE have been developed before our study. However, the majority of these models is not directly comparable to our models. Some were derived in subpopulations that only included certain types of cancer,^6^^,^^14^ whereas other studies predict only catheter-associated VTE,^33^ only DVT,^34^ do not report AUC as a performance measure,^35^ or predict VTE only in hospitalized instead of ambulatory patients.^36^

Hence, only the previously derived EHR-CAT RAM is comparable to our study in most aspects.^15^ This RAM is based on electronic health records from US health care systems. In contrast to our cohort, it includes only patients with newly diagnosed cancer (74.7% in our cohort) and uses the start of systemic therapy as the index date (study inclusion in our study). Furthermore, cerebral venous thrombosis and splanchnic thrombosis were excluded from the analysis. Patients were also followed for such events in our study, but only a few patients experienced these outcomes (within 6 months: 4.0%). Finally, the resulting model was used to expand the Khorana score^7^ by two cancer-specific and four patient-specific predictors for VTE. In total, the study included almost 90 000 individuals between 2011 and 2020 in the derivation and validation data and was then validated again in >300 000 patients.^16^ The resulting AUC in these data sets ranged from 0.68 to 0.72 but were reported at only 0.55 in a cohort study,^17^ which might be explained by the different data sources (electronic health records versus cohort study). In our study, LGR as the best performing model reached an AUC of 0.66. These results may indicate a similar performance threshold for machine learning models in the prediction of cancer-associated VTE. Importantly, the lacking external validation of our model should be considered when interpreting the results.

Therefore, our results together with those of previous studies might show the limitations of machine learning approaches based on currently available cohort study data in the prediction of cancer-associated thrombosis. Both the EHR-CAT RAM, which includes many thousands of patients,^15^ and our study, including a vast amount of potential predictors of VTE, were able to outperform the Khorana score. However, models based on traditional statistical methods developed after the Khorana score have demonstrated increased performance. As an example, the Vienna-CATScore, which is based on only three parameters, has shown AUC in derivation and validation cohorts of 0.62-0.68, which is similar to the reported values of machine learning models.^17^^,^^31^^,^^37^ Previous studies have not directly compared the Vienna-CATScore to machine learning models, but our analysis has demonstrated comparable AUCs between machine learning models and the Vienna-CATScore. Given that our models did not significantly outperform existing risk assessment tools—despite the use of many more parameters for prediction—there appears to be limited clinical benefit of implementing complex machine learning algorithms in their current state. Even among the many markers available in our study, there might be none of sufficient specificity for thrombosis. Other biological states such as inflammation and malignant activity might elevate these markers and obscure risk distinctions. Previous models might already have extracted much of the useful signals for prediction of VTE, and machine learning might therefore struggle to outperform them. Furthermore, cancer-associated VTE is likely influenced by time-dependent parameters to some extent. Factors such as tumor biology, inflammation, treatment, and decreased mobility are not well captured at study inclusion, seemingly adding stochastic noise to predictions based on baseline data. Taking these factors into account, external validation was not pursued in this study, which would be required to demonstrate the robustness of our predictions across different patient populations. We also did not seek to develop a score for incorporation into clinical practice (i.e. calculator or point score). Rather than expecting increased predictive performance solely due to the application of machine learning models, further efforts advancing machine learning-based prediction models, for example, methods enabling competing risk analyses such as Fine and Gray models in traditional statistics, might therefore be warranted. Future approaches may need to leverage high-dimensional data, such as imaging or longitudinal measurements, and integrate them with clinical and laboratory findings to fully harness the potential of machine learning algorithms.

Our study also analyzed performance measures apart from AUC (Table 2). Similar to the Khorana score and the Vienna-CATScore, the specificity and the negative predictive value were generally high, whereas sensitivity and PPV were low.^7^^,^^31^ This is also reflected by the decrease in accuracy after adjustment for imbalance between patients with (8.5%) and without VTE, which decreased from 80%-90% to 50%-60%. These results underline the higher performance of RAMs in ruling out thromboses rather than in confirming them. Unfortunately, these measures are not reported in the EHR-CAT model. Further investigations focusing on models designed to detect thrombosis are therefore warranted.

Furthermore, we conducted an analysis of VTE events at 2 years to detect potential changes in performance and predictive parameters over time but found similar results to the 6-month analysis (Table 2, Figure 1, and Supplementary Figure S3, available at https://doi.org/10.1016/j.esmoop.2026.107764). This might reflect the fact that most VTE occurred during the first 6 months (124 of 186, 66.7%) and could indicate that the additional benefit of predicting VTE in patients with cancer during such intervals might be limited. We also found similar performance metrics in the subgroup of patients with lung cancer, demonstrating validity of our results in a more homogenous group of patients that were previously reported to have a high risk of VTE.^7^^,^^8^^,^^31^

Finally, we compared the input parameter importance between machine learning and traditional statistics. We assessed coefficients of the LGR model and compared them to the associations in a TWAS with VTE at 6 months (Figures 1 and 2). The TWAS is a novel method that is similar to genome-wide association studies and is based on univariable LGR models with correction for multiple testing.^32^ This should not be confused with the LGR machine learning model, which is based on multivariable LGR and includes machine learning-specific methods like class balancing, hyperparameter tuning, and feature selection. In the TWAS, the strongest associations with VTE were found for factor VIII, D-dimer, and sP-selectin, whereas these factors were not associated with VTE in the machine learning LGR. Instead, factor V Leiden and the tumor types rectum, testicular, and pancreas were most strongly associated in the machine learning LGR. A strong negative association was observed for mutations in factor XIII and prothrombin, the tumor type kidney, skin metastases, and for a medical history of chronic heart failure or dyslipidemia. These associations may be surprising but could be explained by class imbalance (only few patients had potentially prothrombotic gene mutations) or the lacking incorporation of competing risk in the analysis, which is currently not feasible in machine learning classification. A similar pattern was also observed in an analysis of VTE at 2 years (Supplementary Figure S3, available at https://doi.org/10.1016/j.esmoop.2026.107764). This indicates that machine learning might be able to detect different patterns for the prediction of VTE, but this does not seem to enable enhanced risk prediction in comparison to traditional statistics. Furthermore, for the reasons discussed above, these associations should not be overinterpreted. Specifically, no causal conclusions should be drawn from these associations, as the goal of our analysis was purely predictive.

This study has several limitations. First, we chose a cut-off of 45% availability of data points in each variable for inclusion into the analysis. This is slightly below the 50% cut-off which was assessed previously,^38^ but only four variables (tumor grading, Karnofsky index, MTHFR 677C/T, Prothrombin 20210G/A) had between 45% and 50% of data points available, whereas all other variables had >50%. Specifically, the variables tumor grading and Karnofsky index were intriguing to the authors, and therefore this cut-off was selected. Many variables in Vienna-CATS were collected only within certain time-limited subprojects (i.e. measurements began after the main study had already started). As a result, missing data were assumed to be missing at random, because the absence of values was due to whether a subproject was active, not because of the variable values themselves. However, this and the generally high number of missing values might have decreased the accuracy of imputations and the performance of the models. Second, the performance comparison with traditional RAMs is limited. All three traditional models (Khorana, Khorana Vienna expansion, and Vienna-CATScore) could not be applied to the entire cohort because for certain cancer sites the models provide no risk assessment (e.g. brain tumors in Vienna-CATScore). The information on RBC growth factor usage was not recorded in Vienna-CATS. This variable was therefore omitted from calculation of the Khorana score, which might have influenced performance assessment of this score. However, the score’s performance was repeatedly reported within the range of our analysis in previous external validation studies.^17^^,^^39^, ^40^, ^41^ Third, the method of deriving AUC differs between the machine learning models and the traditional models. The AUC is based on 100-fold cross-validation in machine learning, whereas calculation is based on the trapezoidal rule in the three traditional models, which might decrease comparability of AUC and CI. Fourth, the cohort in this analysis largely overlaps with the cohort that was also used to develop the eponymous Vienna-CATScore^31^ and the Vienna expansion of the Khorana score.^8^ These results should therefore be interpreted with caution. Finally, data from the Vienna-CATS study was collected until 2019. Recently described biomarker candidates for cancer-associated VTE, such as certain oncogenic mutations or circulating tumor DNA,^42^, ^43^, ^44^, ^45^ might contribute to more accurate predictions but could not be analyzed in the framework of this study because they were not available.

In conclusion, machine learning models based on an extensive set of clinical variables and biomarkers did not substantially improve the prediction of cancer-associated VTE compared with existing RAMs. Future research should therefore focus on exploring new biomarkers, implementing dynamic risk assessment, or leveraging high-level data to enhance predictive performance and clinical utility.
