# Supplementary material for: Prediction of cancer-associated thrombosis by machine learning: results from the Vienna Cancer and Thrombosis Study
Source: ESMO Open. 2026 May 27;11(6):107764. doi: 10.1016/j.esmoop.2026.107764 (PMC13235454; doi:10.1016/j.esmoop.2026.107764)
Supplement: Supplementary Tables S1 and S2 and Figures S1-S3 [file mmc1.docx]

Supplementary Material - “Prediction of cancer-associated thrombosis by machine learning: Results from the Vienna Cancer and Thrombosis Study”

Supplementary Tables

Table S1: Input variables at baseline for VTE prediction

| **Predictor** | **Missing** | **Value** | **Unit** |
| --- | --- | --- | --- |
| Sex | 0 (0.0%) |  |  |
| male |  | 1,171 (53.4%) |  |
| female |  | 1,022 (46.6%) |  |
| Age | 0 (0.0%) | 62 (52 - 68) | years |
| BMI | 4 (0.2%) | 25 (22 - 28) | kg/m² |
| Blood type | 483 (22.0%) |  |  |
| A |  | 683 (39.9%) |  |
| O |  | 654 (38.2%) |  |
| B |  | 262 (15.3%) |  |
| AB |  | 111 (6.5%) |  |
| Rh blood group | 527 (24.0%) |  |  |
| positive |  | 1,342 (80.6%) |  |
| negative |  | 324 (19.4%) |  |
| Smoking | 356 (16.2%) |  |  |
| never |  | 915 (49.8%) |  |
| current |  | 583 (31.7%) |  |
| previous |  | 339 (18.5%) |  |
| Newly diagnosed | 0 (0.0%) | 1,639 (74.7%) |  |
| Tumor type | 0 (0.0%) |  |  |
| lung |  | 397 (18.1%) |  |
| brain |  | 336 (15.3%) |  |
| breast |  | 331 (15.1%) |  |
| lymphoma |  | 271 (12.4%) |  |
| colon |  | 199 (9.1%) |  |
| prostate |  | 161 (7.3%) |  |
| pancreas |  | 156 (7.1%) |  |
| other |  | 66 (3.0%) |  |
| stomach |  | 65 (3.0%) |  |
| multiple myeloma |  | 53 (2.4%) |  |
| kidney |  | 49 (2.2%) |  |
| urogenital |  | 48 (2.2%) |  |
| hepatobiliary |  | 21 (1.0%) |  |
| testicular |  | 18 (0.8%) |  |
| esophageal |  | 16 (0.7%) |  |
| rectum |  | 6 (0.3%) |  |
| Grading | 1,187 (54.1%) |  |  |
| G2 |  | 477 (47.4%) |  |
| G1 |  | 444 (44.1%) |  |
| G3 |  | 85 (8.4%) |  |
| TNM staging | 1,012 (46.1%) |  |  |
| 4 |  | 626 (53.0%) |  |
| 2 |  | 214 (18.1%) |  |
| 3 |  | 185 (15.7%) |  |
| 1 |  | 151 (12.8%) |  |
| 0 |  | 5 (0.4%) |  |
| Karnofsky index | 1,204 (54.9%) | 90.0 (70.0 - 100.0) | % |
| Cancer history | 0 (0.0%) | 172 (7.8%) |  |
| Family history of cancer | 0 (0.0%) | 1,029 (46.9%) |  |
| Adrenal metastasis | 0 (0.0%) | 31 (1.4%) |  |
| Bone metastasis | 0 (0.0%) | 104 (4.7%) |  |
| Brain metastasis | 0 (0.0%) | 106 (4.8%) |  |
| Kidney metastasis | 0 (0.0%) | 5 (0.2%) |  |
| Liver metastasis | 0 (0.0%) | 193 (8.8%) |  |
| Lung metastasis | 0 (0.0%) | 109 (5.0%) |  |
| Lymph node metastasis | 0 (0.0%) | 74 (3.4%) |  |
| Peritoneal metastasis | 0 (0.0%) | 42 (1.9%) |  |
| Skin metastasis | 0 (0.0%) | 7 (0.3%) |  |
| Other metastasis | 0 (0.0%) | 42 (1.9%) |  |
| History of VTE | 0 (0.0%) | 103 (4.7%) |  |
| Family history of VTE | 0 (0.0%) | 181 (8.3%) |  |
| Arterial hypertension | 0 (0.0%) | 740 (33.7%) |  |
| CHF | 0 (0.0%) | 24 (1.1%) |  |
| CKD | 0 (0.0%) | 3 (0.1%) |  |
| CVD | 0 (0.0%) | 222 (10.1%) |  |
| Diabetes | 0 (0.0%) | 198 (9.0%) |  |
| Dyslipidemia | 0 (0.0%) | 116 (5.3%) |  |
| Gastrointestinal disease | 0 (0.0%) | 56 (2.6%) |  |
| Pulmonary disease | 0 (0.0%) | 132 (6.0%) |  |
| Lipidlowering agents | 292 (13.3%) | 238 (12.5%) |  |
| Antiplatelet use | 292 (13.3%) | 281 (14.8%) |  |
| Antithrombin | 55 (2.5%) | 105.0 (94.0 - 115.0) | % |
| APTT | 21 (1.0%) | 33.4 (30.9 - 36.5) | s |
| CRP | 612 (27.9%) | 0.5 (0.2 - 1.7) | mg/L |
| D-dimer | 92 (4.2%) | 0.7 (0.4 - 1.4) | mg/L |
| E-selectin | 164 (7.5%) | 15.4 (10.2 - 22.1) | ng/mL |
| Factor V Leiden | 1,067 (48.7%) |  |  |
| wild type |  | 1,046 (92.9%) |  |
| heterozygous |  | 78 (6.9%) |  |
| homozygous |  | 2 (0.2%) |  |
| Fibrinogen | 30 (1.4%) | 380.0 (312.0 - 466.0) | mg/dL |
| Fibrinogen 455G/A | 941 (42.9%) |  |  |
| wild type |  | 713 (56.9%) |  |
| heterozygous |  | 450 (35.9%) |  |
| homozygous |  | 89 (7.1%) |  |
| FVIII activity | 96 (4.4%) | 189.0 (146.0 - 241.0) | IU/dL |
| FXIII Val34Leu | 991 (45.2%) |  |  |
| wild type |  | 689 (57.3%) |  |
| heterozygous |  | 446 (37.1%) |  |
| homozygous |  | 67 (5.6%) |  |
| Growth differentiation factor 15 | 662 (30.2%) | 1,004.0 (654.0 - 1,752.0) | pg/mL |
| Hematokrit | 24 (1.1%) | 38.9 (35.7 - 41.5) | % |
| Hemoglobin | 9 (0.4%) | 13.1 (11.8 - 14.1) | g/dL |
| Homocystein | 991 (45.2%) | 10.4 (8.5 - 13.2) | Âµmol/L |
| ICAM-1 | 151 (6.9%) | 107.5 (77.7 - 151.1) | ng/mL |
| L-selectin | 152 (6.9%) | 1,332.9 (965.8 - 1,837.2) | ng/mL |
| Leukocytes | 9 (0.4%) | 7.2 (5.7 - 9.6) | G/L |
| Lp(a) | 662 (30.2%) | 13.0 (7.0 - 41.0) | mg/dL |
| Lymphocytes | 410 (18.7%) | 1.4 (1.0 - 1.8) | G/L |
| MCH | 19 (0.9%) | 29.9 (28.5 - 31.0) | pg |
| MCHC | 13 (0.6%) | 33.7 (32.9 - 34.3) | g/dL |
| MCV | 15 (0.7%) | 88.5 (85.3 - 91.6) | fL |
| Monocytes | 414 (18.9%) | 0.5 (0.4 - 0.7) | G/L |
| MTHFR 677C/T | 1,169 (53.3%) |  |  |
| heterozygous |  | 480 (46.9%) |  |
| wild type |  | 428 (41.8%) |  |
| homozygous |  | 116 (11.3%) |  |
| Neutrophils | 414 (18.9%) | 4.8 (3.6 - 6.6) | G/L |
| NT-proBNP | 662 (30.2%) | 108.0 (54.0 - 230.0) | pg/mL |
| P-selectin | 168 (7.7%) | 18.8 (14.4 - 27.1) | ng/mL |
| PAI | 75 (3.4%) | 2.5 (1.0 - 6.3) | ng/mL |
| Platelet volume | 47 (2.1%) | 10.2 (9.6 - 10.8) | G/L |
| Platelets | 9 (0.4%) | 250.0 (199.0 - 310.0) |  |
| Prothrombin 20210G/A | 1,120 (51.1%) |  |  |
| wild type |  | 1,043 (97.2%) |  |
| heterozygous |  | 30 (2.8%) | pmol/L |
| Prothrombin fragment 1 + 2 | 58 (2.6%) | 226.0 (165.0 - 320.0) | % |
| Quick | 258 (11.8%) | 105.0 (92.0 - 121.0) | T/L |
| RBC | 21 (1.0%) | 4.4 (4.0 - 4.7) | % |
| RDW | 16 (0.7%) | 13.8 (13.1 - 14.6) | ng/mL |
| sLOX-1 | 117 (5.3%) | 15.0 (7.1 - 32.9) | ng/mL |
| Soluble P-selectin | 36 (1.6%) | 37.9 (29.0 - 49.0) | nM |
| TGA (peak) | 10 (0.5%) | 346.7 (202.6 - 513.7) | mg/dL |
| VCAM-1 | 153 (7.0%) | 161.8 (104.5 - 233.3) | ng/mL |
| n = 2,193 | | | |

Only parameters in the Vienna Cancer and Thrombosis Study with < 55% missing value were used for the analysis. APTT = activated partial thromboplastin time, BMI = body mass index, CHF = congestive heart failure, CKD = chronic kidney disease, CRP = C-reactive protein, CVD = cardiovascular disease, GDF = growth differentiation factor, MCH = mean corpuscular hemoglobin, MCHC = mean corpuscular hemoglobin concentration, MCV = mean corpuscular volume, PAI = plasminogen activator inhibitor-1, RBC = red blood cells, RDW = red cell distribution width, TGA = thrombin generation assay, VTE = venous thromboembolism.

Table S2: Model performances for venous thromboembolism prediction in lung cancer patients at six months.

| **Model** | **Accuracy** | **Sensitivity** | **Specificity** | **PPV** | **NPV** | **BAcc** | **AUC** |
| --- | --- | --- | --- | --- | --- | --- | --- |
| LGR | 0.74 (0.74 - 0.75) | 0.43 (0.40 - 0.45) | 0.76 (0.75 - 0.77) | 0.08 (0.08 - 0.09) | 0.96 (0.96 - 0.96) | 0.59 (0.58 - 0.61) | 0.62 (0.61 - 0.63) |

Numbers in brackets represent the 95% confidence interval. AUC = area under the curve (C-statistics), BAcc = balanced accuracy, LGR = Logistic Regression.

Supplementary Figures


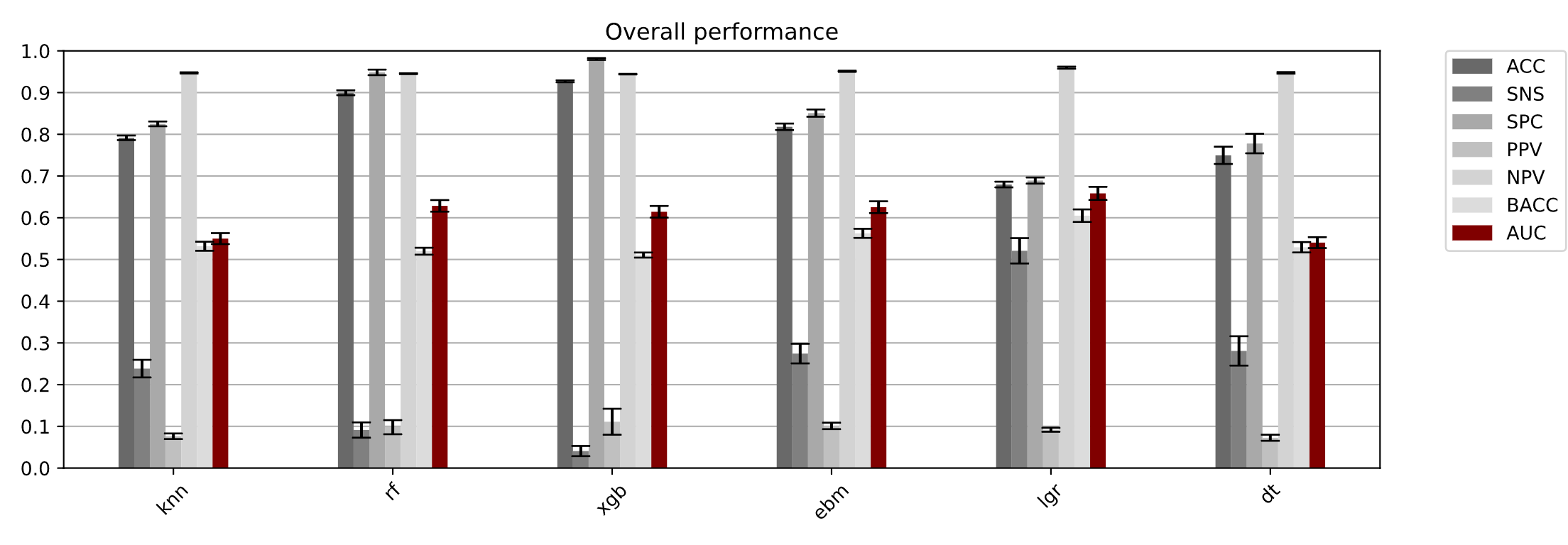


Figure S1: Performance of machine learning models for the prediction of venous thromboembolism within 6 months. Error bars represent the 95% confidence interval. AUC = area under the receiver operating characteristic curve (AUC), ACC = accuracy, SNS = sensitivity, SPC = specificity, PPV = positive predictive value, NPV = negative predictive value, BACC = balanced accuracy, knn = K-Nearest Neighbor, rf = Random Forest, xgb = Extreme Gradient Boosting, ebm = Explainable Boosting Machine, lgr = Logistic Regression, dt = Decision Tree.


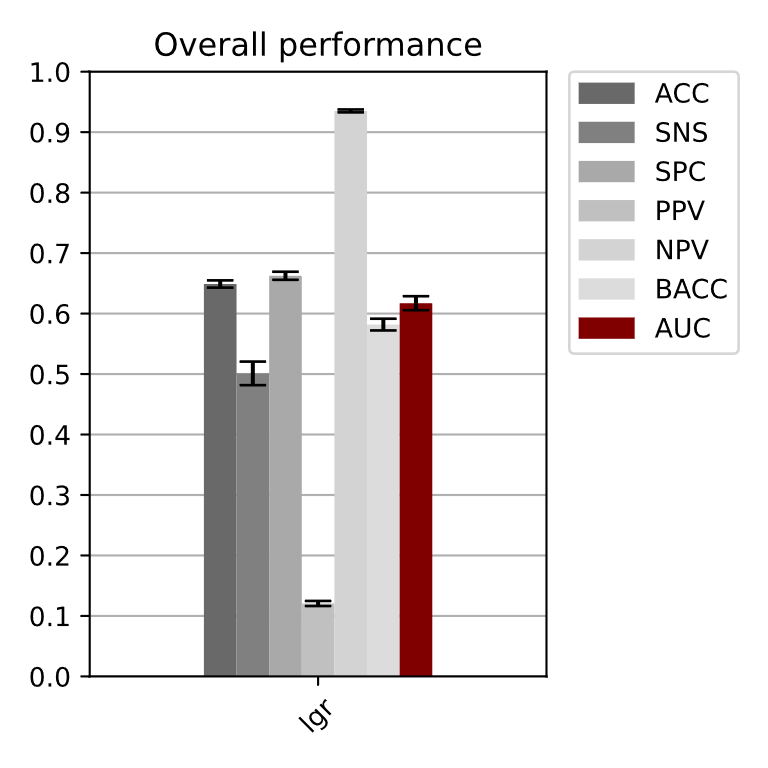


Figure S2: Performance of Logistic Regression for the prediction of cancer-associated thrombosis within 2 years. Error bars represent the 95% confidence interval. AUC = area under the receiver operating characteristic curve (AUC), ACC = accuracy, SNS = sensitivity, SPC = specificity, PPV = positive predictive value, NPV = negative predictive value, BACC = balanced accuracy, lgr = Logistic Regression.


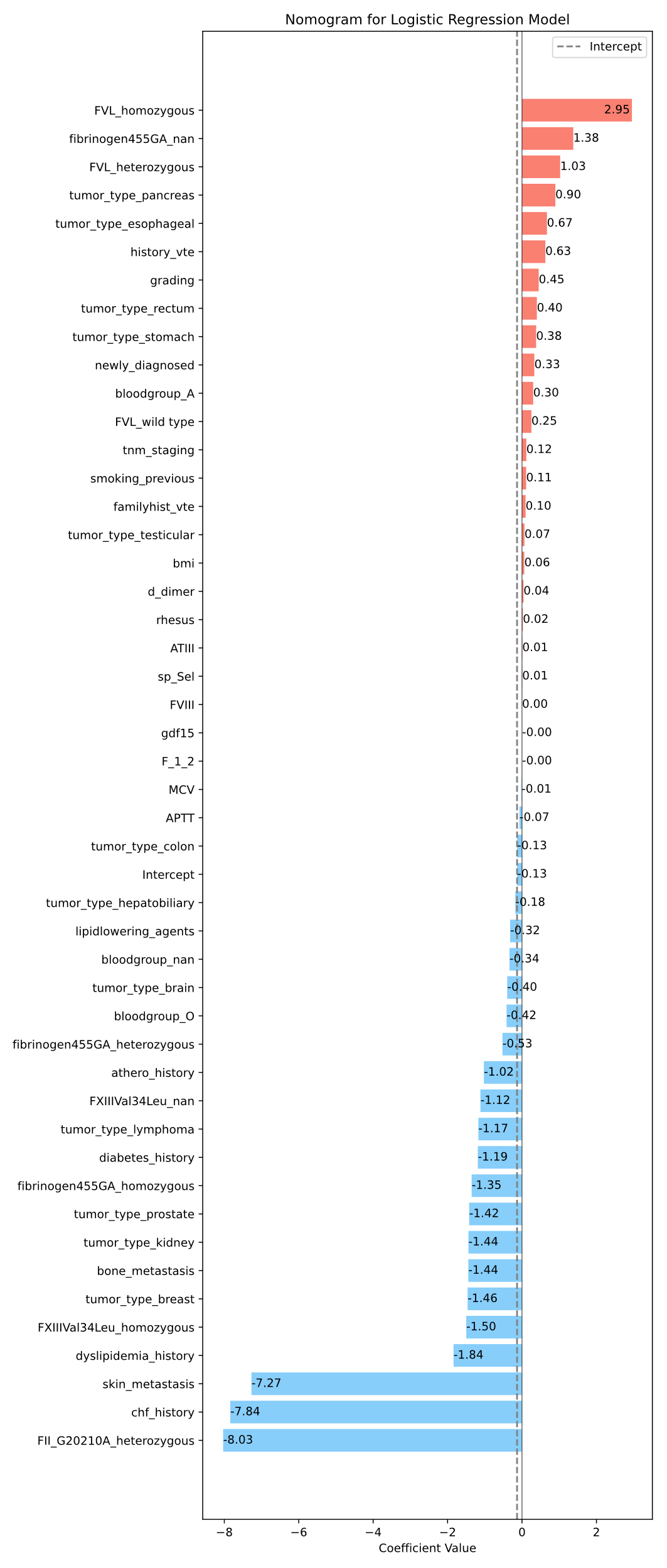


Figure S3: Coefficients of prediction variables in Logistic Regression for the prediction of cancer-associated thrombosis at 2 years.
